# Long-Term Occupational Exposure to Organic Solvents Affects Color Vision, Contrast Sensitivity and Visual Fields

**DOI:** 10.1371/journal.pone.0042961

**Published:** 2012-08-15

**Authors:** Thiago Leiros Costa, Mirella Telles Salgueiro Barboni, Ana Laura de Araújo Moura, Daniela Maria Oliveira Bonci, Mirella Gualtieri, Luiz Carlos de Lima Silveira, Dora Fix Ventura

**Affiliations:** 1 Instituto de Psicologia, Universidade de São Paulo, São Paulo, Brazil; 2 Núcleo de Neurociências e Comportamento, Universidade de São Paulo, São Paulo, Brazil; 3 Departamento de Fisiologia, Universidade Federal do Pará, Belém, Brazil; 4 Núcleo de Medicina Tropical, Universidade Federal do Pará, Belém, Brazil; University of Sussex, United Kingdom

## Abstract

The purpose of this study was to evaluate the visual outcome of chronic occupational exposure to a mixture of organic solvents by measuring color discrimination, achromatic contrast sensitivity and visual fields in a group of gas station workers. We tested 25 workers (20 males) and 25 controls with no history of chronic exposure to solvents (10 males). All participants had normal ophthalmologic exams. Subjects had worked in gas stations on an average of 9.6±6.2 years. Color vision was evaluated with the Lanthony D15d and Cambridge Colour Test (CCT). Visual field assessment consisted of white-on-white 24–2 automatic perimetry (Humphrey II-750i). Contrast sensitivity was measured for sinusoidal gratings of 0.2, 0.5, 1.0, 2.0, 5.0, 10.0 and 20.0 cycles per degree (cpd). Results from both groups were compared using the Mann–Whitney U test. The number of errors in the D15d was higher for workers relative to controls (p<0.01). Their CCT color discrimination thresholds were elevated compared to the control group along the protan, deutan and tritan confusion axes (p<0.01), and their ellipse area and ellipticity were higher (p<0.01). Genetic analysis of subjects with very elevated color discrimination thresholds excluded congenital causes for the visual losses. Automated perimetry thresholds showed elevation in the 9°, 15° and 21° of eccentricity (p<0.01) and in MD and PSD indexes (p<0.01). Contrast sensitivity losses were found for all spatial frequencies measured (p<0.01) except for 0.5 cpd. Significant correlation was found between previous working years and deutan axis thresholds (rho = 0.59; p<0.05), indexes of the Lanthony D15d (rho = 0.52; p<0.05), perimetry results in the fovea (rho = −0.51; p<0.05) and at 3, 9 and 15 degrees of eccentricity (rho = −0.46; p<0.05). Extensive and diffuse visual changes were found, suggesting that specific occupational limits should be created.

## Introduction

In Brazil, vehicle fuelling is done by workers employed by gas stations exclusively to fill car tanks. This type of job typically requires an 8-hour shift where the worker is chronically exposed to solvents and other toxic substances found in gasoline, diesel and ethanol fuels. Exposure occurs by breathing vapors exhaled by fuels and absorption through the eyes [1]. Ethanol fuel is composed of ethanol (92.6% to 93.8%) and water (6 to 8%) [2]. On the other hand, gasoline and diesel sold in Brazil contain a mixture of organic solvents (mostly comprised of benzene, toluene and xylene) predominantly destined to octane boosting [3], [4]. Furthermore, Brazilian automotive gasoline receives an addition of 20% anhydrous ethanol [5]. Although a similar mixture of anhydrous ethanol and gasoline is sold in other countries, we did not find data on the pharmacokinetics of such mixture. The neurotoxic nature of benzene, toluene, xylene and ethanol is well known, and the harm due to occupational exposure (even below occupational limits) to those substances has been suggested by several publications [6], [7], [8]. Despite this, few studies have been carried out to evaluate the effects of chronic solvent exposure in gas station workers [9], [10], and very little is known about the harmfulness of these solvent mixtures.

Overall, 29,843,665 m^3^ of gasoline, 49,239,039 m^3^ of diesel and 15,074,300 m^3^ of ethanol were sold in Brazilian gas stations during the year 2010. In the state of São Paulo where the present study was performed, there are approximately 8,817 gas stations [11]. Despite the great number of employees, there are no specific occupational exposure limits for gas station workers in Brazil.

The relationship between organic solvent exposure and visual loss has been previously studied. Early evaluation of visual function usually reveals subclinical effects on subjects' vision [12], [1]. Such results may help to establish safer occupational exposure limits [12], [1], [8]. Color vision is frequently studied in this field of research, and the arrangement methods are usually the chosen tests [1] done under diverse lighting conditions and using different analysis procedures, which may account for conflicting results in different studies [8]. The use of more than one color vision test may help clarify the severity of the visual losses. Comparing the results of panel tests such as FM100, D15 and D15d with results of computerized tests is one method that has been used [13], [14], [15].

Losses in achromatic contrast sensitivity associated with exposure to solvents have been confirmed by different evaluation techniques such as psychophysical contrast sensitivity chart tests [16], [17], visual-evoked potentials [18], [19], computerized spatial and temporal contrast sensitivity tests [9] and a letter contrast sensitivity test [20]. Visual field losses have also been detected in individuals exposed to solvents, both with the Goldman perimeter [19] and with Humphrey static automated perimetry [9]. Although Kiyokawa et al. [19] reported central losses, Lacerda et al. [9] found visual field constriction.

The limited number of studies and diversity of results in the literature suggest that an effort for increasing the precision of testing procedures is needed. The present work is aimed at investigating the existence of visual impairment caused by occupational exposure to a mixture of solvents, focusing on both chromatic and achromatic vision.

Based on the existing literature, one would expect a direct action of the solvents on the retina, thalamus and cortex as a whole because organic solvents are highly liposoluble [6]. With respect to color vision, there is a suggestion that the blue–yellow system may be more sensitive to the effects of organic solvent intoxication [1], [8]. Short-wavelength sensitive cones are reported by some authors to be more sensitive to toxic substances than the other cone types [21], [22]. However, other authors show similar red–green and blue–yellow losses in cases of occupational solvent intoxication [1], [9].

In the early 20^th^ century, Köllner suggested that acquired color vision losses will first be of the blue–yellow type (reflecting damage to the outer retina) and only later evolve to a red–green type (reflecting damage to the inner retinal layers and optic nerve). Although many exceptions to Köllner's rule are now well described (e.g., in glaucoma [23]), occupational neuroscientists evoke Köllner's rule to interpret the severity and time-course of the neurotoxic exposures behind the color vision defects [24], [21], [9]. The argument is that the acquired color vision loss will evolve to a red–green defect only after starting as a blue–yellow defect and that moderate exposure profiles would result in isolated blue–yellow defects, whereas severe exposure profiles would affect both red–green and blue–yellow discrimination. Considering the chronic nature of the solvent exposure experienced by gas station workers, we could expect both blue–yellow and red–green color vision loss in our results.

For the achromatic vision tests, contrast sensitivity loss is expected at all spatial frequency ranges because the liposolubility of the solvents suggests nonselective, widespread neural alterations at all levels of the central nervous system. For the same reason, diffuse losses are also expected in the perimetry. Finally, we hypothesize that visual losses will be correlated to the number of years working in this occupation because longer exposures would result in cumulative damage to the nervous system.

## Materials and Methods

Participants underwent an individual semi-structured anamnesis interview prior to visual function evaluation and a complete ophthalmologic examination. History of smoking, alcohol or substance abuse and previous diagnosis of any neurological or psychiatric diseases were exclusion criteria for both patients and controls. Four participants were excluded due to smoking history. None of the participants were taking any type of medication known to affect the central nervous system. The ophthalmologic examination was done before the testing battery and inclusion criteria were absence of ophthalmologic pathologies, absence of posterior subcapsular cataract and maximum of grade 1 cortical opacity (C1), nuclear color (NC1) and nuclear opalescence according to the LOCSIII classification system for lens opacity. All participants had normal or corrected to normal visual acuity (Snellen 20/20).

We tested 25 workers (20 males aged 36.4±8.9 years) and 25 controls with no history of chronic exposure to toxic substances (10 males aged 33.8±8.8 years). All workers were employed for at least 12 months by gas stations operating under the supervision of the Brazilian National Petroleum Agency (ANP) at the time of the test. The average number of previous working years in gas stations for the group of workers was 9.64±6.19 years and represented 8-hour shifts, 6 days/week. Overall, the workers claimed that they did not use personal protective gear (masks, gloves or similar) during the work shift. Excluded from the sample were all workers who worked in fuel distribution prior to 1992 when Brazilian fuel contained lead and methanol. All tests were performed in one eye randomly chosen. The contralateral eye was kept occluded during the tests. The entire test battery lasted an average of less than 2 h. The test sequence was randomized for each participant in order to avoid the interference of fatigue on the results.

Written informed consent was obtained from all participants, and procedures were approved by the local ethics committee (Institute of Psychology, University of São Paulo, Brazil, Project 2008.061) in accordance with international guidelines.

### 2.1. Color vision tests

Color vision was assessed with the Lanthony D15d test and the Cambridge Colour Test 2.0 (Cambridge Research Systems). The Lanthony D15d is a desaturated adaptation of the Farnsworth D15 and is comprised of 15 chips covered by Munsell papers of fixed saturation and variable hues. Each chip has 1.5 degrees of visual angle when viewed at a distance of 50 cm. The test was performed under standardized lighting conditions (two Sylvania Octron 6500K FO32W/65K fluorescent lamps; illuminance = 1.49×10^3^ lux). The task of the subject was to order the caps, according to hue, in three consecutive trials. Only the last trial was considered for data analysis. The results were presented as a Color Confusion Index (CCI), a value obtained dividing the participant's score by the normal score [25].

The Cambridge Colour Test (CCT) is a computerized test [26] that proved to be an efficient tool in the detection and quantification of acquired color vision defects [13], [14], [27], [28], [29], [30], [31], [32]. The test uses pseudoisochromatic stimuli and a four-alternative forced choice psychophysical procedure, avoiding eventual biases known to occur in arrangement tests and other classes of color vision tests. The test stimulus (presented through a VSG 2/5 Visual Stimulus Generator in a Viewsonic G90fB 19″ CRT monitor) consists of a pseudoisochromatic stimulus in which the background is presented at a given chromaticity and the target, an adapted “Landolt C”, is presented with a different chromaticity. The stimulus prevents the interference of spatial and luminance cues in the discrimination by using a mosaic of spatial and luminance noise to compose the stimulus. The stimulus is composed of circles of varying sizes (5.7 to 22.8 arcmin) and luminances between 7 and 15 cd/m^2^. The Landolt C gap appears to the subject in one of four positions: right, left, up or down. The subject's task was to report the position of the gap using a remote control. The gap measures 1.25° visual angle and the stimulus has a diameter of 7.5° visual angle. A four-alternative forced choice psychophysical staircase was used, starting at specific points of the CIE 1976 diagram and extending to the center of the color space (u′v′: 0.197 & 0.469). From one trial to the next, the chromaticity of the target approached the background chromaticity after every correct response and moved away from it after every incorrect response. The test ended after 11 response reversals for each tested axis of the CIE 1976 color space. Thresholds were estimated using the last six reversals for each axis.

Two testing protocols were used in the CCT. The Trivector, a fast screening test whose results are not biased by learning or fatigue effects [29], measured thresholds for color discrimination in the protan, deutan and tritan confusion axes. The Ellipses Test measured a MacAdam ellipse from the discrimination thresholds estimated in eight vectors of the CIE color space around a background chromaticity (u′v′ = 0.197, 0.469), the same used in the Trivector test. The area of the ellipse was used as an indicator of sensitivity (smaller area, higher sensitivity) and the ratio between the larger and smaller ellipse diameters (ellipticity) was used as an indicator of specificity of defect (larger ellipticity suggests a more specific defect).

### 2.2. Genetic analysis

Genetic analysis was performed to determine the possibility of congenital color vision deficiency in three out of four subjects with elevated color discrimination thresholds in the CCT Trivector test. DNA was extracted from blood samples using a purification kit (PUREGENE DNA, Gentra System). To evaluate the presence and position of L and M genes in the array of the X chromosome, a first genetic experiment was performed according to Neitz & Neitz [33]. To exclude the presence of polymorphisms in the L and M genes, genetic sequencing was carried out. Polymerase chain reaction (PCR) was performed to amplify exons 2, 3 and 4 of both L and M genes. Primers and PCR conditions used were the same as described by Neitz et al. [34]. A blood sample of a normal trichromat subject was used as a negative control in all genetic experiments. In addition, for positive control, blood samples from a protanope and a deuteranope subject (subjects EL and RT, respectively) were also included.

### 2.3. Achromatic vision tests

Achromatic visual processing was evaluated using the Metropsis software (Cambridge Research Systems) for contrast sensitivity and the Humphrey II-750i Perimeter (Carl Zeiss Meditec) for white-on-white automated perimetry. The Metropsis software used a video resolution of 1280×1024 pixels and average luminance of 34.4 cd/m^2^. Vertical achromatic sinusoidal gratings of 0.2, 0.5, 1.0, 2.0, 5.0, 10.0 and 20.0 cpd were used as stimuli. A two-alternative forced choice psychophysical procedure was used, and the subject's task was to identify, using a remote control response box, whether the stimulus was presented at the left or right side of the screen. A dynamic psychophysical staircase with logarithmic steps was used. At the beginning of the test, the stimulus contrast decreased 1.5 dB after every three consecutive correct responses. After the first incorrect response, the staircase started a more strict criteria and the stimulus contrast increased by 1.0 dB after every incorrect response and decreased 0.7 dB after every three consecutive correct responses. The staircase design followed guidelines from Garcia-Pérez [35]. The session ended after eight response reversals were recorded for each grating tested. The thresholds were calculated averaging the contrast values of the response reversals for each grating, and contrast sensitivity was estimated as the reciprocal of the threshold values. The subject's viewing distance for the Metropsis and Cambridge Colour Test was 300 cm.

The 24–2 white-on-white central protocol of the Humphrey Field Analyzer II–750i was used to evaluate sensitivity throughout the visual field. The stimuli consisted of white light spots measuring approximately 0.43° visual angle (Goldmann III size), presented randomly for 200 ms in the fovea and in another 52 locations within the central 21° of the visual field against a white background with average luminance of 10 cd/m^2^ inside a Ganzfeld stimulator. All stimuli were presented with an initial intensity of 30 dB. The viewing distance for this test was 30 cm. The subject's task was to press the button on a remote control whenever a light spot was perceived. The subject was instructed to keep the gaze at the same fixation point during the entire test and a video camera was used to control the subject's fixation throughout the test. The psychophysical procedure consisted of a simple staircase in which every correct response decreased the stimulus intensity in that location by 2 dB and every response omission would result in a 4 dB increase of stimulus intensity. A threshold value was determined for each location after two response reversals. In order to minimize testing time and to maximize reliability of the results, the SITA standard algorithm (Swedish Interactive Threshold Algorithm) was used [36], [37]. The test displays three indices of sensitivity throughout the visual field: 1) threshold values for each location measured, 2) mean deviation (MD) and 3) the pattern standard deviation (PSD). MD and PSD are values derived according to the Humphrey system that illustrate to what extent the patient's results deviate from the normal. MD scores are more sensitive to diffuse defects, whereas PSD scores are more sensitive to localized visual field defects [38].

Statistical analysis was performed with the software Statistica 9.0 (Statsoft Inc.). For each data set, a complete descriptive analysis with normal distribution verification by Kolmogorov-Smirnov and Shapiro-Wilk tests were done. Mann-Whitney U test was used for all intergroup comparisons and Spearman's rank correlation coefficient was used to check correlations between previous number of working years and visual performance for all tests.

## Results

Scores for all tests and demographic data for both groups are summarized in Table 1.

**Table 1:** Average results for both groups in all tests.

	WORKERS		CONTROL GROUP	
	Mean	SD	Mean	SD
Age (years)	36.40	8.91	33.80	8.80
Number of working years	9.64	6.19	–	–
Trivector protan	63.38	35.57	39.50	12.53
Trivector deutan	76.25	72.70	38.00	13.11
Trivector tritan	97.79	42.38	58.68	18.91
Elipse área	878.83	539.79	533.09	211.92
D15d CCI	1.48	0.42	1.06	0.08
Perimetry fovea	37.17	1.97	37.05	1.47
Perimetry 3°	32.67	1.94	33.49	1.00
Perimetry 9°	31.42	1.65	32.53	0.85
Perimetry 15°	28.49	2.13	29.78	0.95
Perimetry 21°	27.54	3.99	29.83	1.25
Perimetry MD	−1.80	2.16	−0.30	0.94
Perimetry PSD	2.62	1.90	1.81	0.46
CSF 0.2 cpd	21.61	9.37	47.49	14.77
CSF 0.5 cpd	65.08	20.46	70.47	19.11
CSF 1.0 cpd	109.55	40.50	144.51	37.66
CSF 2.0 cpd	120.84	52.83	261.24	115.97
CSF 5.0 cpd	60.35	28.80	211.53	118.44
CSF 10.0 cpd	21.45	17.84	71.75	43.97
CSF 20.0 cpd	2.52	0.77	11.47	8.10

SD, standard deviation of the mean.

**Figure 1:**
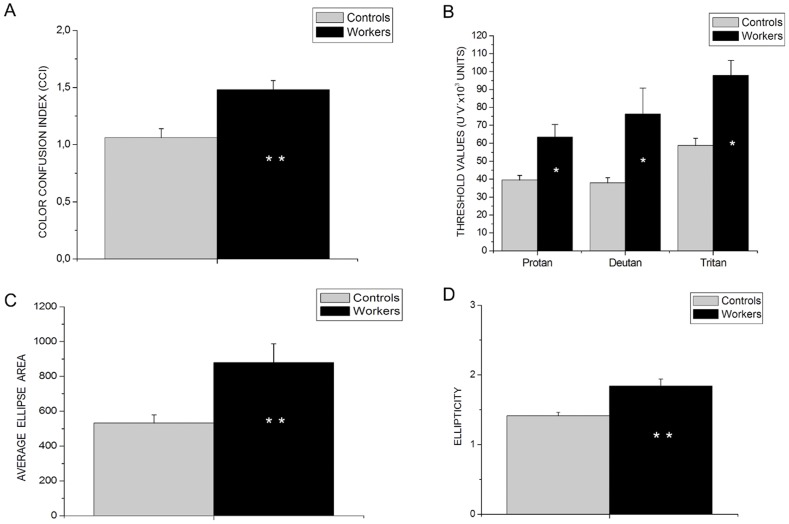
Results for color vision tests. (A) Color Confusion Indexes for both groups. (B) Threshold values for both groups in the confusion lines measured with CCT Trivector test. (C) Average ellipse area measured with the CCT. (D) Average ellipticity measured with the CCT Ellipse Test. Asterisks denote significant visual loss of the group of workers. *p<0.05; **p<0.01. Vertical bars represent standard errors.

In the CCT Trivector test, workers showed higher thresholds in protan (U = 121; p<0.01), deutan (U = 87; p<0.01) and tritan (U = 84; p<0.01) axes compared to controls (see Figure 1B). Higher values were also found for area (U = 156; p<0.05) and ellipticity (U = 147; p<0.01) on the ellipses test in comparison to controls (see Figures 1C and 1D). For the Lanthony D15d, group color confusion indexes for workers were higher than those for the control group (U = 43; p<0.01; see Figure 1A). For the protan, deutan and tritan axes thresholds, respectively, 37.5%, 33.28% and 45.76% of the workers had results higher than the 95% confidence intervals (CIs) of the control group: 33.28% of the workers had average ellipse area values higher than the 95% CIs of the control group, 70.7% of the workers had CCI values higher than the 95% CIs of the control group.

Four subjects had high Trivector thresholds suggesting a congenital color vision deficiency (see subjects JDA, EFS, APL and ANC in Table 2). Three out of four subjects with very elevated Trivector thresholds were genotyped to exclude congenital color vision losses. Genetic analysis was not performed for subject EFS due to failure to contact the subject. Group comparisons done excluding subject EFS showed that our results did not change and visual losses found remained equally significant. Results from genetic analyses showed that the three workers examined had a normal opsin gene array in the X chromosome with L gene in the first position and the M gene downstream. Sequences of L and M genes of the subjects were in accordance with the normal trichromat subject control sample (see Table 3). In the positive controls, the protanope subject had no L gene as expected but showed a normal M gene sequence, whereas for the deuteranope subject the reverse was true: absence of M gene with normal sequence of L gene.

**Table 2:** Demographic information, visual acuity and Trivector results for each worker tested using color vision tests.

Worker	Age	Acuity	Years of Work	Protan	Deutan	Tritan	CCI
URS	19	20/12.5	1	40	60	112	1.06
LHA	21	20/12.5	1	57	42	62	1.15
AMNM	25	20/16	5	42	35	86	1.06
MSR	25	20/12	6	47	36	55	2.63
RMS	26	20/16	1	42	48	94	1.44
ESB	27	20/25	10	85	66	78	1.35
ASC	30	20/12.5	4	51	48	81	1.40
AOF	32	20/15	10	74	51	122	1.19
FBA	33	20/20	3	42	44	62	1.04
ESSC	35	20/12.5	1	44	45	88	1.16
UJS	35	20/20	12	29	51	61	1.16
ALSB	37	20/20	5	29	40	73	1.24
FCS	37	20/12.5	16	63	76	98	1.48
ALS	38	20/20	3	64	46	72	1.64
JDA	40	20/20	14	120	147	104	1.95
HTM	40	20/20	17	45	45	71	1.29
MES	42	20/20	8	80	65	116	1.26
JML	44	20/16	16	40	46	64	2.01
JGP	44	20/16	16	67	82	76	1.37
EFS	44	20/16	12	72	116	80	1.10
JLS	46	20/20	11	57	48	129	1.40
APL	45	20/20	18	198	393	259	1.53
CBA	47	20/16	17	44	54	122	1.89
ANC	48	20/20	18	102	144	129	2.32
JEP	50	20/16	16	50	78	151	1.98
**Workers Mean**	**36.40**	**.**	**9.64**	**63.38**	**76,25**	**97.79**	**1.48**
**Workers SD**	**8.91**	**.**	**6.19**	**35.57**	**72,70**	**42.38**	**0.42**
**Control Mean**	**33.80**	.	.	**39.50**	**38.00**	**58.68**	**1.06**
**Control SD**	**8.80**	.	.	**12.53**	**13.11**	**18.91**	**0.08**

SD, standard deviation of the mean.

**Table 3:** Results of the genetic analysis for three workers, a normal trichromat as negative control, and two color defective subjects: a protanope (EL) and a deuteranope (RT) as positive controls (description of L and M gene sequences).

Subject	L gene			M gene		
	Exon 2	Exon 3	Exon 4	Exon 2	Exon 3	Exon 4
	65 111 116	153 171 174 178 180	230 233 236	153 171 174 178 180	153 171 174 178 180	230 233 236
APL	T I S	L V V I S	I A M	I V Y	M V A I A	T S V
JDA	T I S	L V A I S	I A M	I V Y	M V V I A	T S V
ANC	T I S	L V A I S	I A M	I V Y	M V V I A	T S V
RT	T I S	L V A I S	I A M	NP	NP	NP
EL	NP	NP	NP	I V Y	M V A I A	T S V
Control	T I S	L V A I S	I A M	I V Y	M V A I A	T S V

Significant correlations with number of previous years of work were found for deutan thresholds (rho = 0.59; p<0.05). The Color Confusion Index (rho = 0.52; p<0.05) suggested that a longer working history resulted in a poorer visual performance.

**Figure 2:**
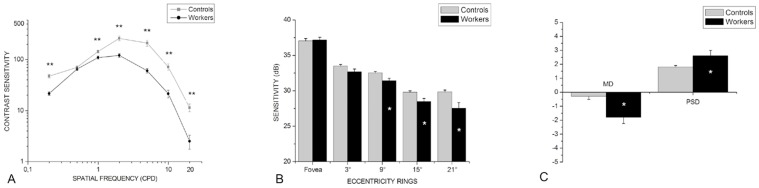
Results for achromatic tests. (A) Contrast sensitivity curves for both groups. (B) Sensitivity values for the fovea and eccentricity rings measured by the perimetry. (C) Average MD and PSD perimetry values. Asterisks denote significant visual loss in the group of workers. *p<0.05; **p<0.01. Vertical bars represent standard errors.

Figure 2A shows that for the spatial contrast sensitivity test, the workers group was significantly less sensitive than controls at all spatial frequencies measured [0.2 cpd (U = 22; p <0.01), 1.0 cpd (U = 101; p<0.01), 2.0 cpd (U = 46.5; p<0.01), 5.0 cpd (U = 44; p<0.01), 10.0 cpd (U = 44; p<0.01), 20 cpd (U = 9; p<0.01)] except for 0.5 cpd (U = 247; p = 0.37). Of the workers, 68%, 20%, 28%, 40%, 44%, 36%, and 48% had sensitivity values below the 95% CIs of the control group for frequencies of 0.2, 0.5, 1.0, 2.0, 5.0, 10.0 and 20.0, respectively. The number of working years exposed to solvents and contrast sensitivity performance were not significantly correlated.

When analyzing perimetry data, only reliable fields were considered. Whenever a participant had a number of fixation losses, false positives or false negatives that exceeded the normative values of the Humphrey perimeter, the test was interrupted. Nine of 25 participants had to interrupt and repeat the examination. The workers group had a significant loss of sensitivity at 9° (U = 118.5; p<0.01), 15° (U = 113; p<0.01) and 21° (U = 85; p<0.01) eccentricity rings when compared to the control group (see Figure 2B). MD (U = 97.5; p<0.01) and PSD (U = 102; p<0.01) were significantly different between groups (see Figure 2C): 36% and 28% of the workers had scores outside the 95% CIs of the control group for MD and PSD indexes, respectively. The eccentricity ring analysis showed 12%, 28%, 40%, 28% and 40% workers below the 95% CIs of the control group for the fovea, 3°, 9°, 15° and 21° eccentricity rings, respectively.

Significant negative correlations between number of previous years of work and perimetry data were found for the fovea (rho = −0.51; p<0.05), 3° (rho = −0.46; p<0.05), 9° (rho = −0.46; p<0.05) and 15° (rho = −0.46; p<0.05) eccentricity rings. This implies that a longer working history is correlated with lower sensitivity. No significant correlations were found between number of previous working years and MD or PSD values.

Workers' age was significantly correlated with the number of previous years of work (rho = 0.77; p<0.05). To assess age as a possible confounding factor, we evaluated whether the age of the control group is significantly correlated to the scores. Deutan threshold in the control group was not correlated (rho = 0.28; p = 0.2), whereas the Lanthony D15d CCI was significantly correlated with age (rho = 0.67; p<0.05). For perimetry data of the control group, foveal sensitivity was significantly correlated with age (rho = −0.47; p<0.05). All eccentricity ring thresholds, MD and PSD were not correlated with age (−0.03<rho<−0.24; 0.21<p<0.88).

In total, the results suggest extensive and diffuse visual system changes in both chromatic and achromatic discriminations. Losses in deutan discrimination and visual field were significantly correlated with the number of working years, suggesting that a longer working history leads to greater visual losses.

## Discussion

Several studies in regard to the relationship between organic solvent exposure and color vision loss report visual changes exclusively at the blue–yellow confusion axis as shown by Muttray et al. [21] and in numerous works reviewed by Paramei et al. [8]. Our results for the CCT suggest both red–green and blue–yellow losses and the Lanthony D15d suggest a diffuse color vision loss as well. These findings are in accordance with the reports of Päällysaho et al. [39] and Zavalic et al. [40] for populations with distinct solvent exposure profiles. Our results are similar to Lacerda et al. [9] who reported a significant and diffuse color vision loss using the Farnsworth-Munsell 100 and an adaptation of the CCT in a sample of gas station workers.

A diffuse color vision loss such as the one presented here has been regarded as indicative of a severe intoxication profile [9], [21], [24]. When compared to the organic solvent concentration present in other contexts (i.e., solvents in the printing industry or organic solvent abuse), solvent concentrations present in fuels may be considered moderate (values for gasoline: toluene <3%, benzene <2%, xylene <2.5% [41]). Also, the fact that deutan thresholds and CCIs are significantly correlated with working time is notable. This suggests that the chronic nature of the exposure may constitute a severe exposure profile for this job.

In this context, age cannot be excluded as a possible confounding factor because age and previous number of working years are highly correlated (rho = 0.77; p<0.05). To evaluate the significance of this confounding factor, we assessed whether the age of the control group was significantly correlated with the scores in the comparisons where the workers group scores were significantly correlated with previous working years. We found that among these, only CCI and foveal sensitivity of the control group were significantly correlated with age. Therefore, significant correlations between perimetry results and deutan thresholds with previous working years are indicative of the neurotoxic exposure effects and not a result of age-related visual system changes.

The correlations presented here partially replicate the existing literature. Semple et al. [42] found a positive significant correlation between number of previous working years and color vision loss in a sample of dockyard workers occupationally exposed to a mixture of organic solvents. Lacerda et al. [9] found low, nonsignificant correlations between number of previous working years and scores of diverse visual tests in a sample of gas station workers. The differences in results found here and in the study of Lacerda et al. [9] suggest that the time course of solvent-induced occupational visual losses deserves further investigation.

The possibility of a contamination of mild congenital color defective patients in our sample with the effects of solvent exposure was examined in cases of subjects with extremely elevated color discrimination thresholds. Generally, protan or deutan congenital color defective patients have normal thresholds in the tritan range. This was not the case for participants ANC, EFS, APL or JDA, a fact suggesting that they were not congenital color vision deficient. There was, however, a possibility that congenital color vision defects were combined with acquired color vision losses, causing elevated thresholds at all axes. Genetic analysis resolved the issue, establishing that the high color discrimination thresholds were not caused by congenital factors. This finding reinforces the idea that this particular profile of solvent intoxication may cause extensive color vision losses.

Our contrast sensitivity results are in accordance with Boeckelmann & Pfister [16], although they partially disagree with Lacerda et al. [9] and Schaper et al. [43]. Boeckelmann & Pfister [16] reported loss of sensitivity to gratings of 1.5, 3.0, 6.0 and 12.0 cpd in a group of 42 printers occupationally exposed to a mixture of solvents. In contrast, Schaper et al. [43] reported no contrast sensitivity loss in a similar sample and Lacerda et al. [9] reported a loss of sensitivity only for high spatial frequencies (20 and 30 cpd). The sensitivity loss in low and medium frequency bands supports the hypothesis that this change in contrast sensitivity is due to alterations in the central nervous system and not in the optical media of the eye.

These differences may be attributed in part to differences in methodology among studies. Boeckelmann & Pfister [16] and Schaper et al. [43] used different chart tests (Vistech VCTS 6500 and Pelli-Robson Contrast Sensitivity Chart, respectively), whereas Lacerda et al. [9] used a computerized test similar to ours, although adopting a “Yes-No” psychophysical procedure. In comparison to a chart test, a computerized contrast sensitivity test allows more precise stimuli presentation and the implementation of more rigorous psychophysical procedures.

Overall, our perimetry results are in agreement with the data from other authors [9], [19]. Lacerda et al. [9] found a similar change in MD and PSD values along with a loss of sensitivity from 10° of eccentricity towards the periphery. The fact that both MD and PSD showed significant changes suggests a diffuse effect of the exposure on the visual field of the workers. Qualitative analyses of the visual field results also suggest a diffuse visual field loss. On the other hand, analysis of sensitivity according to the eccentricity did not show significant changes at the fovea or 3° ring. The fact that Humphrey's 24–2 perimetry has a smaller resolution in that area may influence these results. The test presents five stimuli within the central 3° of the visual field and 48 stimuli altogether between 3° and 21° eccentricity rings. On the other hand, of all eccentricity rings, fovea had the highest correlation with the number of previous working years (rho = −051; p<0.05) and the foveal sensitivity was significantly correlated with all eccentricity rings (0.50<rho<0.64; p<0.05). Also, because the central portion of the visual field has the highest spatial resolution acuity, contrast sensitivity losses at 20 and 30 cpd may reflect a central visual field loss. Not using smaller targets in the perimetry (we used the perimeter's third largest target due to its clinical popularity) may conceal subclinical central visual field losses due to the high acuity of this area. Together the above-mentioned arguments suggest that one cannot interpret our results as indicative of visual field constrictions.

The present results of chromatic and achromatic vision tests suggest the involvement of different visual mechanisms in the sensory changes reported. It is known that the integrity of the contrast sensitivity response relies primarily on the parvocellular visual pathway for high spatial frequencies and on the magnocellular visual pathway for low spatial frequencies [44], [45]. Because the workers group showed equivalent contrast sensitivity losses at all spatial frequency bands tested, there are reasons to believe that occupational exposure to the mixture of solvents present at a gas station site would affect both magno- and parvocellular function. Furthermore, red–green and blue–yellow discrimination appear to be mediated mostly by parvo- and koniocellular visual pathways, respectively [46], [47]. The diffuse loss found in the Lanthony D15d arrangement performance, together with the similar protan, deutan and tritan losses, plus the large ellipse areas with moderate ellipticity found in the CCT imply red–green and blue–yellow losses. This suggests that the occupational exposure profile studied here may similarly affect parvo- and koniocellular function.

As pointed out by Gobba and Cavalleri [1], the pathogenesis of occupational visual loss caused by organic solvents is still unclear. Some of the most popular hypotheses are as follows: (i) visual pathway degeneration related to axonopathy [48], (ii) direct influence of the solvent on photoreceptor function [1], [22], and (iii) cortical (and/or retinal) changes in neurotransmitter systems like glutamate [49], dopamine [50] and acetylcholine [51] as reviewed by Eisenberg [7]. Contemporary research in regard to organic solvent-induced visual losses suggests a predominant neural origin of the defects because ocular changes are generally moderate or nonexistent [1], [9], [52]. Parvo-, magno- and koniocellular pathways link the retina to the primary visual cortex via the lateral geniculate body. However, considering the data presented here, it is not possible to clearly state whether visual losses found are due to retinal or cortical changes (or possibly both).

Electroretinography (ERG) should be implemented in future studies to evaluate the existence of any alterations at the retinal level that may account for the psychophysical losses reported in the literature. Most of the electrophysiological evaluations in this field are limited to visual-evoked potentials. The generalized visual losses found with the above-mentioned hypotheses, the reports of organic solvent-related cognitive alterations [53] and the well-known liposoluble nature of solvents such as toluene, benzene and xylene offer a strong argument for a generalized mechanism of action of organic solvents on the visual system.

Visual changes found with our results are significant and robust, although there is substantial variability in the workers' group performance. We tested workers with different ages (19 to 50 years) from different gas stations, with different chronic exposure history (1 to 18 years) and with possible genetic differences influencing the solvent metabolism. Gene polymorphisms may modulate solvent biotransformation and affect the outcomes of neurotoxic exposures [54]. However, Semple et al. [42] showed that three gene polymorphisms that affect biotransformation did not influence the extent of color vision loss induced by occupational solvent exposures.

The variability in the data supports the need to use more than one testing procedure when evaluating the visual outcome of occupational neurotoxic exposures [13], [15], [28], [31], [32]. Visual system performance and cognitive changes are not straightforward and may unpredictably affect the testing performance. Malingering may also be a concern under some circumstances. Therefore, using complementary tests that require different tasks may prevent false positives. Because variability is not uncommon in this field of research, the use of complementary tests may help interpret the results.

In summary, our results suggest that occupational exposure to a mixture of solvents by gas station workers may impair visual function in many aspects. Changes in parvo-, magno- and koniocellular pathway functions are suggested by the present data along with significant correlations between the number of previous working years and sensory loss. The results presented here are in agreement with several other studies suggesting that chronic exposure to organic solvents leads to broad functional and structural changes in the central nervous system [1], [55], [56]. We have no direct measures of solvent exposure, but the results presented here strongly suggest that gas station workers are exposed to sufficiently high solvent levels that may induce changes in nervous system function. Specific occupational limits/exposure standards for gas station workers should be created.
